# Effects of Dexmedetomidine Infusion on Inflammatory Responses and Injury of Lung Tidal Volume Changes during One-Lung Ventilation in Thoracoscopic Surgery: A Randomized Controlled Trial

**DOI:** 10.1155/2018/2575910

**Published:** 2018-04-05

**Authors:** Chun-Yu Wu, Yi-Fan Lu, Man-Ling Wang, Jin-Shing Chen, Yen-Chun Hsu, Fu-Sui Yang, Ya-Jung Cheng

**Affiliations:** ^1^Anesthesiology Department, National Taiwan University Hospital, Taiwan; ^2^Anesthesiology Department, National Taiwan University Hospital, Hsinchu Branch, Taiwan; ^3^Surgery Department, National Taiwan University Hospital, Taipei, Taiwan

## Abstract

One-lung ventilation in thoracic surgery provokes profound systemic inflammatory responses and injury related to lung tidal volume changes. We hypothesized that the highly selective a2-adrenergic agonist dexmedetomidine attenuates these injurious responses. Sixty patients were randomly assigned to receive dexmedetomidine or saline during thoracoscopic surgery. There is a trend of less postoperative medical complication including that no patients in the dexmedetomidine group developed postoperative medical complications, whereas four patients in the saline group did (0% versus 13.3%, *p* = 0.1124). Plasma inflammatory and injurious biomarkers between the baseline and after resumption of two-lung ventilation were particularly notable. The plasma high-mobility group box 1 level decreased significantly from 51.7 (58.1) to 33.9 (45.0) ng.ml^−1^ (*p* < 0.05) in the dexmedetomidine group, which was not observed in the saline group. Plasma monocyte chemoattractant protein 1 [151.8 (115.1) to 235.2 (186.9) pg.ml^−1^, *p* < 0.05] and neutrophil elastase [350.8 (154.5) to 421.9 (106.1) ng.ml^−1^, *p* < 0.05] increased significantly only in the saline group. In addition, plasma interleukin-6 was higher in the saline group than in the dexmedetomidine group at postoperative day 1 [118.8 (68.8) versus 78.5 (58.8) pg.ml^−1^, *p* = 0.0271]. We conclude that dexmedetomidine attenuates one-lung ventilation-associated inflammatory and injurious responses by inhibiting alveolar neutrophil recruitment in thoracoscopic surgery.

## 1. Introduction

One-lung ventilation (OLV) is mandatory during thoracic surgery, but it may provoke profound systemic inflammatory responses that participate in the development of lung injury [1–3]. Alveolar neutrophil recruitment, induced by several proinflammatory mediators such as high-mobility group box 1 (HMGB1) [4, 5] and monocyte chemoattractant protein 1 (MCP-1) [6], is one of the major sources of OLV-induced inflammatory responses [1, 7, 8]. Moreover, atelectasis during OLV and lung redistension after resumption of two-lung ventilation are additional mechanisms to promote neutrophil sequestration in the lung and aggravate inflammatory responses [1, 9]. Proinflammatory mediators, such as plasma neutrophil elastase, and anti-inflammatory mediators, such Clara cell protein (CC16), related to these lung tidal volume changes also play potential roles in OLV-induced inflammatory responses [10, 11].

Dexmedetomidine (DEX), a highly selective alpha-2 agonist with sedative and analgesic properties, is well-known not only for its ability to preserve respiratory function but also for its anti-inflammatory effect that has been described in various studies including the experimental lung injury model [12] and clinical studies conducted in cardiac surgery with cardiopulmonary bypass [13] and laparoscopic surgery [14]. However, the protective effects of DEX infusion during thoracic surgery on the aforementioned injurious responses remain less clear. In this study, we investigated whether intraoperative DEX infusion alleviates the expression of OLV-induced injurious mediators in thoracoscopic surgery.

## 2. Materials and Methods

This double-blind, single-institution, prospective trial was approved by the Research Ethics Committee of National Taiwan University Hospital and was registered at http://clinicaltrials.gov with the identifier NCT02439905. Patients undergoing elective thoracoscopic surgery between July 2015 and June 2016 were enrolled. Patients with the following conditions were excluded: active infection status, defined as WBC count > 10,000 or body temperature > 38.3°C; abnormal liver or kidney function, defined as liver aminotransferase > 100 mg.dl^−1^ or serum total bilirubin >2 mg.dl^−1^; estimated glomerular filtration rate < 60 ml.min^−1^ per 1.73 m^2^; and abnormal cardiopulmonary function, such as patients with heart failure beyond New York Hearth Association Functional Classification class II, chronic obstructive pulmonary disease, or active coronary arterial disease.

We obtained written informed consent from all patients on the day before surgery, which was performed by an investigator who was unaware of the randomization result. On arrival at the operating theatre, patients were allocated to the study arms in a 1 : 1 ratio according to a computer-generated randomization list, which an independent statistician prepared before the trial. Then patients were divided into two groups, namely the saline group (receiving equal amount of saline infusion; *N* = 30) and the DEX group (receiving 0.5 *μ*g.kg^−1^.h^−1^ DEX infusion throughout surgery; *N* = 30). This dose was chosen based on most clinical studies administrating DEX as an adjuvant to general anesthesia [15] except omitting loading dose to avoid hemodynamic instability [16]. Each patient received general anesthesia induced by infusing fentanyl 1.5–2.5 mcg.kg^−1^, propofol 2 mg.kg^−1^, glycopyrrolate 0.2 mg, and rocuronium 1 mg.kg^−1^ and infusion of experimental medication. After general anesthesia, OLV was conducted by using a double-lumen tracheal tube or bronchial blocker. During surgery, the patients in both groups were maintained with sevoflurane to control the bispectral index between 40 and 60. The treatment protocols with the same respiratory and hemodynamic care protocols were applied to each patient. For example, mechanical ventilation was set with a tidal volume: 8 ml.kg^−1^ for two-lung ventilation and 5 ml.kg^−1^ for OLV with a positive end-expiratory pressure of 5 cmH_2_O; the respiratory rate was titrated to maintain the end-tidal CO_2_ between 30 and 40 cmH_2_O. The intraoperative fraction of inspired oxygen was initially set with an air :  oxygen ratio at 1 : 1 during two-lung ventilation and titrated to maintain S_p_O_2_ > 94% during OLV. After surgery completion, we recruited two-lung ventilation via manual cyclic bagging with a pressure of 30 cmH_2_O lasting for 2 minutes. Hemodynamics were controlled to maintain a mean arterial pressure of >55 mmHg and a heart rate between 50 and 100 bpm by intravenous boluses of ephedrine or labetalol.

Plasma levels of biomarkers, including HMGB1, MCP-1, interleukin-6 (IL-6), plasma neutrophil elastase, and CC16, were measured and compared between the baseline (T_1_, after anesthesia induction), 1 h after resumption of two-lung ventilation from OLV (T_2_), and postoperative day 1 (T_3_). Serum concentrations of HMGB1 (Chondrex Inc., Redmond, WA, USA), MCP-1 (BioLegend, San Diego, CA, USA) and IL-6 (BioLegend, San Diego, CA, USA), plasma neutrophil elastase (Hycult Biotech, Uden, The Netherlands), and CC16 (BioVendor LLC, Candler, NC, USA) were measured using enzyme-linked immunosorbent assay kits.

### 2.1. Statistical Analyses

Among the investigated plasma biomarkers, HMGB1 has the most potent proinflammatory efficacy [17]. Therefore, we calculated that a sample size of 17 patients in each group was required to detect an absolute 30% decrease in plasma HMGB1 level, with a power of 0.8 and *p* = 0.05 considered significant based on a previous report [13]. A Fisher exact test or chi-square test was employed to analyze dichotomous data, the Student *t*-test was used for normally distributed continuous data, and the Mann–Whitney *U* test was used for nonparametric ordinal data. Repeated measures analysis of variance with the group and time factors, followed by post hoc analysis with the Tukey's test, was used to compare serially measured variables. Statistical analyses were performed using MedCalc software (MedCalc Inc., Mariakerke, Belgium).

## 3. Results

**Figure 1:**
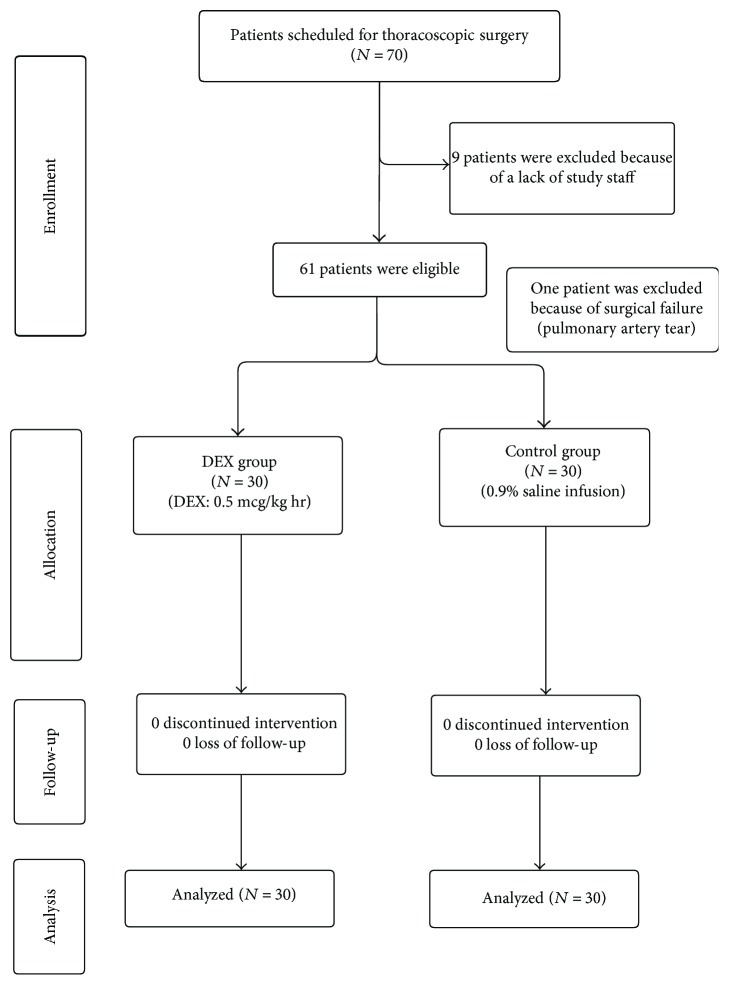
CONSORT diagram.

Figure 1 shows the CONSORT diagram of inclusion. A total of 70 patients met the inclusion criteria and agreed to attend this trial. Nine patients were excluded due to clinical complications. One patient in the saline group dropped out because of safety concerns due to a pulmonary artery tear during the operation.

The demographic characteristics were similar between the two groups (Table 1). More patients in the DEX group had primary pulmonary malignancy (83.3% versus 96.7%; *p* = 0.1945; Table 1) as well as more advanced cancer staging (M1: 0% versus 20.0%; *p* = 0.0252; stage III or IV: 23.3% versus 46.6%; *p* = 0.0641; Table 1).

Intraoperative profiles were also comparable between the two groups, including the surgical type, amount of blood loss, OLV duration, and administrated fluid amount, with the exception that patients in the DEX group received less fentanyl (Table 2). However, compared with patients in the saline group, those in the DEX group revealed a larger intraoperative mean arterial pressure range with comparable highest mean arterial pressure but lower lowest mean arterial pressure [62.7 (7.2) versus 58.3 (7.1) mmHg in saline and DEX groups, respectively, *p* = 0.0193] as well as slower intraoperative heart rate range (Table 2).

Postoperative outcomes are summarized in Table 3. Although there was an increase in surgical complications, particularly subcutaneous emphysema (2 versus 8 patients in the saline and DEX groups, respectively; *p* = 0.0395; Table 3), patients in the DEX group tended to have more favorable hospital outcomes; this included lower incidence (16.7% versus 33.3%, *p* = 0.2326) and shorter length (0.2 ± 0.6 versus 0.8 ± 1.7 day, *p* = 0.1152) of intensive care unit stay as well as a general lack of postoperative medical complications. By contrast, four patients developed medical complications in the saline group (13.3% versus 0%; *p* = 0.1124; Table 3).

**Figure 2:**
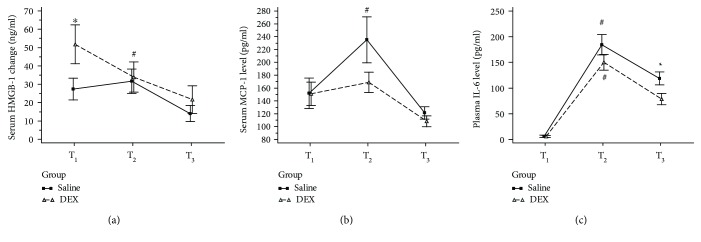
Changes in perioperative plasma levels of one-lung ventilation-induced inflammatory biomarkers. (a). Perioperative changes in plasma high-mobility group box 1 protein levels. ^∗^ indicates a higher level in the DEX group than in the saline group with a *p* < 0.05 at T_1_. # indicates an intragroup increase between T_1_ and T_2_ with *p* < 0.05 in the DEX group. (b). Perioperative changes in plasma monocyte chemoattractant protein 1 levels. # indicates an intragroup increase between T_1_ and T_2_ with *p* < 0.05 in the saline group. (c). Perioperative changes in plasma interleukin-6 levels. ^∗^ indicates a higher level in the saline group than in the DEX group with *p* < 0.05 at T_3_. # indicates intragroup increases between T_1_ and T_2_ with *p* < 0.05 in both DEX and saline groups.

Changes in plasma inflammatory biomarkers, including HMGB-1, IL-6, and MCP-1, are shown in Figure 2. Attenuated serum inflammatory responses were observed among the DEX group patients: (1) although a significantly higher baseline plasma HMGB1 level (T_1_) was observed in the DEX group, a significant decrease in plasma HMGB1 levels between T_1_ and T_2_ was noted only in the DEX group [from 51.7 (58.1) ng.ml^−1^ to 33.9 (45.0) ng.ml^−1^; *p* < 0.05; Figure 2(a)], which was not observed in the saline group; (2) a significant increase in MCP-1 levels between T_1_ and T_2_ that was observed only in the saline group [151.8 (115.1) to 235.2 (186.9) pg.ml^−1^; *p* < 0.05; Figure 2(b)], which was not observed in the DEX group; and (3) plasma IL-6 level at postoperative day 1 (T_3_) was significantly higher in the saline group than in the DEX group [118.8 (68.8) versus 78.5 (58.8) pg.ml^−1^, *p* = 0.0271; Figure 2(c)].

**Figure 3:**
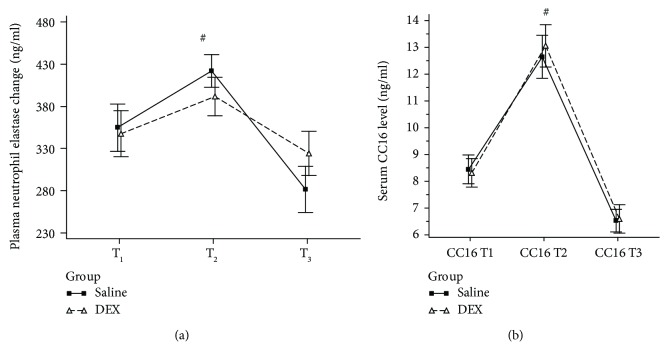
Changes in perioperative plasma levels of one-lung ventilation-induced lung tidal volume injury biomarkers. (a). Perioperative changes in plasma neutrophil elastase levels. # indicates an intragroup increase between T_1_ and T_2_ with *p* < 0.05 in the saline group. (b). Perioperative changes in plasma Clara cell protein levels. # indicates intragroup increases between T_1_ and T_2_ with *p* < 0.05 in both DEX and saline groups.

Changes in plasma biomarkers related to lung tidal volume changes, including plasma neutrophil elastase and CC16, are shown in Figure 3. A significant increase in plasma neutrophil elastase levels between T_1_ and T_2_ was observed only in the saline group [354.8 (154.5) to 421.9 (106.1) ng.ml^−1^; *p* < 0.05; Figure 3(a)] but not in the DEX group. By contrast, plasma CC16 levels significantly increased between T_1_ and T_2_ in both the DEX and saline groups (Figure 3(b)).

## 4. Discussion

The major finding of this study is that intraoperative DEX infusion attenuates OLV-induced injurious responses in patients undergoing thoracoscopic surgery.

In this study, we found that only patients in the DEX group had a significant reduction in plasma HMGB1 levels between the baseline (T_1_) and 1 hour after resumption of two-lung ventilation (T_2_). HMGB1 is present in the nuclei of most mammalian cells. When released into plasma, this protein serves as a danger signal that provokes profound inflammatory responses, regarded as “the nuclear weapon in immune arsenal” [17], in association with the recruitment of neutrophil cells [4] and participates in the development of acute lung injury [18], as well as ventilator-induced lung injury [19, 20]. In addition, the expression levels of other proinflammatory cytokines, including IL-6 and MCP-1, are upregulated by HMGB1 [17, 21–23]. Regarding the effects of intraoperative DEX infusion on the attenuation of these inflammatory responses, Ueki et al. reported DEX infusion to inhibit inflammatory responses gauged by serum HMGB1 and IL-6 in patients undergoing cardiac surgery [13]. Furthermore, Jiang et al. reported that DEX attenuated MCP-1 expression in an experimental study involving lung ischemia-reperfusion injury [12]. Compatible to these findings, our results confirm that the protective effect of DEX infusion is also present in a clinical thoracoscopic scenario. This is clinically relevant because these inflammatory responses are strongly associated with lung injury development, particularly through the important role of MCP-1 in airway neutrophil and macrophage recruitment [24–26]. Because alveolar neutrophils are one of the major sources of lung inflammatory product secretion [7], our result indicates that intraoperative DEX infusion attenuates OLV-induced inflammatory responses through the prevention of neutrophil recruitment.

In addition to the inflammatory effects, HMGB1 plays potential roles in cancer development. HMGB1 is overexpressed in many cancers [27], and plasma HMGB1 levels are elevated in patients with lung cancer [28]. This is consistent with our findings that baseline HMGB1 levels (normally undetectable in plasma) were abnormally high in the patients in both groups. Moreover, the plasma HMGB1 level is associated with cancer TMN staging [29], acting as an extracellular signalling molecule during tumor progression [30]. Because cancer TMN staging remained unknown during patient randomization and could be determined only after surgery, this may explain the difference in baseline HMGB1 levels between the two study groups, because more patients in the DEX group had cancer at an advanced stage. Despite the most operation type in both groups is lobectomy, higher cancer staging still may result in higher surgical complexity. This may be the reason why patients in the DEX group developed more surgery-related complications such as subcutaneous emphysema.

The atelectasis and redistention of lungs from one lung to two lungs during thoracic surgery is another potential mechanism to promote OLV-induced inflammatory responses. We focused on two particular biomarkers, plasma neutrophil elastase and CC16, for analysis in this study. Recruited neutrophils secrete plasma neutrophil elastase in the alveoli to induce lung epithelial apoptosis [31]. In addition, increased plasma neutrophil elastase could be specifically induced by atelectasis injury [10], which is inevitable during OLV. In this study, the increase in plasma neutrophil elastase was observed only in the saline group, but not the DEX group. Therefore, inhibition of neutrophil activation and recruitment by DEX infusion may also attenuate atelectasis-induced injury mediated by plasma neutrophil elastase. By comparison, CC16 is a secreted product of the respiratory epithelium produced primarily within the Clara cells of the distal respiratory and terminal bronchioles of the lung. It plays a role in attenuation of inflammatory responses and, consequently, a higher serum CC16 level is associated with better outcomes in patients with acute respiratory distress syndrome [32]. Furthermore, a rapid increase of serum CC16 has been reported after lung distention injury, possibly to elicit protective effects [10, 33]. In this study, serum CC16 levels were significantly increased between the baseline (T_1_) and 1 hour after resumption of two lung (T_2_) in both groups. This indicates that the protective pathway of CC16 remains intact in patients receiving DEX infusion. Taken together, these findings suggest that intraoperative DEX infusions also elicit protective effects as part of the injurious responses generated during lung atelectasis and redistension in thoracic surgery.

Despite higher dose of DEX may elicit more potent anti-inflammatory responses [34], the benefit of the attenuation of OLV-induced injurious responses for DEX infusion during thoracoscopic surgery should be balanced with the concerns of increased risks of intraoperative hemodynamic instability. In our study, more patients in the DEX group received ephedrine boluses because of their lower mean arterial pressure and slower heart rate. However, the hemodynamic instability may be clinically irrelevant because the postoperative outcomes were comparable between the two groups, while patients in the DEX group showed a trend of more favorable incidence of ICU stay and fewer postoperative medical complications possibly because of attenuated ischemia-reperfusion injury.

This trial has some limitations. First, despite the DEX group having fewer postoperative medical complications, we did not observe a significant improvement in hospital outcomes in this study. This may be because thoracoscopic surgery is associated with fewer major complications than open thoracotomy for lung cancer resection [35], making the benefits of the attenuation of OLV-induced injurious responses more difficult to compare by clinical outcomes. Because of ischemia-reperfusion injury and attenuation, these inflammatory responses could be associated with reduced postoperative complications [36–38], for more complex surgery involving OLV such as esophagectomy, the benefit of intraoperative DEX infusion may be more evident. Second, the effect of postoperative DEX administration was not investigated in this study, because most patients were successfully extubated in the operating theatre and additional sedation was not required. Regarding the benefits of the DEX respiration-sparing properties, investigation of potential anti-inflammatory effects of postoperative DEX administration may be warranted.

## 5. Conclusion

DEX infusion during thoracoscopic surgery effectively attenuates OLV-induced injurious responses by inhibition of neutrophil recruitment.

## Figures and Tables

**Table 1:** Demographics of participants in the two groups.

	Saline (*N* = 30)	DEX (*N* = 30)	*p* value
Age (yr)	58.7 (10.1)	59.0 (8.8)	*p* = 0.9028
Gender (M/F)	16/14	15/15	*p* = 0.7980
Weight (kg)	64.4 (12.2)	64.5 (12.5)	*p* = 0.9763
Operation type (*N*, %)			*p* = 0.5447
Lobectomy	22 (73.3%)	24 (80%)	
Others	8 (27.7%)	6 (20%)	
Comorbidities (*N*, %)			
Hypertension	8 (26.7%)	9 (30%)	*p* = 0.7786
Diabetes	6 (20%)	2 (6.7%)	*p* = 0.1331
Others	15 (50%)	10 (33.3%)	*p* = 0.1933
ASA class (*N*, %)			
I	1 (3.3%)	2 (6.7%)	*p* = 0.5569
II	18 (60%)	14 (46.7%)	*p* = 0.3047
III	11 (36.7%)	14 (46.7%)	*p* = 0.4360
Primary lung malignancy (*N*, %)			*p* = 0.1945
Yes	25 (83.3%)	29 (96.7%)	
No	5 (16.7%)	1 (3.3%)	
Lung cancer staging (*N*, %)			
T			*p* = 0.0151
1	3 (10.0%)	12 (40.0%)	
2	18 (60.0%)	10 (33.3%)	
3	4 (13.3%)	4 (13.3%)	
4	0 (0%)	3 (10.0%)	
N			
0	14 (46.7%)	20 (66.7%)	*p* = 0.5301
1	5 (16.7%)	3 (10.0%)	
2	6 (20.0%)	6 (20.0%)	
M			
0	25 (83.3%)	23 (76.7%)	*p* = 0.0252
1	0 (0%)	6 (20.0%)	
Final staging (*N*, %)			
I	9 (30.0%)	12 (40.0%)	*p* = 0.0641
II	9 (30.0%)	3 (10.0%)	
III-IV	7 (23.3%)	14 (46.6%)	

**Table 2:** Intraoperative profiles.

	Saline (*N* = 30)	DEX (*N* = 30)	*p* value
OLV duration (min)	153.6 (60.0)	157.6 (74.4)	*p* = 0.8225
Fentanyl dosage (mcg)	158.3 (45.6)	132.5 (41.1)	*p* = 0.0248
Blood loss (ml)	84.8 (89.6)	101.7 (154.0)	*p* = 0.6067
Fluid administrated (ml)	1005.0 (377.7)	1108.3 (703.5)	*p* = 0.4812
Mean arterial pressure (mmHg)			
Highest	101.6 (13.3)	100.0 (12.5)	*p* = 0.6259
Lowest	62.7 (7.2)	58.3 (7.1)	*p* = 0.0193
Heart rate (bpm)			
Highest	93.8 (13.8)	87.4 (10.4)	*p* = 0.0470
Lowest	69.9 (9.0)	64.9 (7.1)	*p* = 0.0218
Transfusion needed (*N*, %)	1 (3.3%)	2 (6.7%)	*p* = 1.000
Patients needed ephedrine (*N*, %)	7 (23.3%)	20 (66.7%)	*p* = 0.0016

OLV: one-lung ventilation.

**Table 3:** Postoperative outcomes.

	Saline (*N* = 30)	DEX (*N* = 30)	*p* value
Chest tube duration (day)	3.6 ± 2.6	3.7 ± 2.7	*p* = 0.9610
Surgical complication (*N*, %)	5 (16.7%)	12 (30.0%)	*p* = 0.0840
Air leaks need pleurodesis	1 (3.3%)	3 (10.0%)	*p* = 0.3017
Subcutaneous emphysema	2 (6.7%)	8 (26.7%)	*p* = 0.0395
Chylous drainage	2 (6.7%)	1 (3.3%)	*p* = 0.5491
Medical complication (*N*, %)	4 (13.3%)	0 (0%)	*p* = 0.1124
Pneumonia	1 (3.3%)	0 (0%)	*p* = 0.3198
Cardiovascular	2 (6.7%)	0 (0%)	*p* = 0.1527
Delirium	1 (3.3%)	0 (0%)	*p* = 0.3198
Patients needed ICU (*N*, %)	10 (33.3%)	5 (16.7%)	*p* = 0.2326
ICU stay (day)	0.8 (1.7)	0.2 (0.6)	*p* = 0.1152
Hospital stay (day)	5.9 ± 3.1	5.6 ± 2.5	*p* = 0.6491

ICU: intensive care unit.

## References

[1] Lohser J., Slinger P. (2015). Lung injury after one-lung ventilation: a review of the pathophysiologic mechanisms affecting the ventilated and the collapsed lung.

[2] Olivant Fisher A., Husain K., Wolfson M. R. (2012). Hyperoxia during one lung ventilation: inflammatory and oxidative responses.

[3] García-de-la-Asunción J., García-del-Olmo E., Perez-Griera J. (2015). Oxidative lung injury correlates with one-lung ventilation time during pulmonary lobectomy: a study of exhaled breath condensate and blood.

[4] Huebener P., Pradere J. P., Hernandez C. (2015). The HMGB1/RAGE axis triggers neutrophil-mediated injury amplification following necrosis.

[5] Tadie J. M., Bae H. B., Jiang S. (2013). HMGB1 promotes neutrophil extracellular trap formation through interactions with Toll-like receptor 4.

[6] De Conno E., Steurer M. P., Wittlinger M. (2009). Anesthetic-induced improvement of the inflammatory response to one-lung ventilation.

[7] Engels G. E., van Oeveren W. (2015). Biomarkers of lung injury in cardiothoracic surgery.

[8] Grommes J., Soehnlein O. (2011). Contribution of neutrophils to acute lung injury.

[9] Eguchi T., Hamanaka K., Kondo R. (2014). Lung re-expansion following one-lung ventilation induces neutrophil cytoskeletal rearrangements in rats.

[10] Fernandez-Bustamante A., Klawitter J., Repine J. E. (2014). Early effect of tidal volume on lung injury biomarkers in surgical patients with healthy lungs.

[11] Schilling T., Kozian A., Huth C. (2005). The pulmonary immune effects of mechanical ventilation in patients undergoing thoracic surgery.

[12] Jiang L., Li L., Shen J., Qi Z., Guo L. (2014). Effect of dexmedetomidine on lung ischemia‑reperfusion injury.

[13] Ueki M., Kawasaki T., Habe K., Hamada K., Kawasaki C., Sata T. (2014). The effects of dexmedetomidine on inflammatory mediators after cardiopulmonary bypass.

[14] Kang S.-H., Kim Y.-S., Hong T.-H. (2013). Effects of dexmedetomidine on inflammatory responses in patients undergoing laparoscopic cholecystectomy.

[15] Li B., Li Y., Tian S. (2015). Anti-inflammatory effects of perioperative dexmedetomidine administered as an adjunct to general anesthesia: a meta-analysis.

[16] Ickeringill M., Shehabi Y., Adamson H., Ruettimann U. (2004). Dexmedetomidine infusion without loading dose in surgical patients requiring mechanical ventilation: haemodynamic effects and efficacy.

[17] Lotze M. T., Tracey K. J. (2005). High-mobility group box 1 protein (HMGB1): nuclear weapon in the immune arsenal.

[18] Kim J. Y., Park J. S., Strassheim D. (2005). HMGB1 contributes to the development of acute lung injury after hemorrhage.

[19] Ogawa E. N., Ishizaka A., Tasaka S. (2006). Contribution of high-mobility group box-1 to the development of ventilator-induced lung injury.

[20] Wolfson R. K., Mapes B., Garcia J. G. N. (2014). Excessive mechanical stress increases HMGB1 expression in human lung microvascular endothelial cells via STAT3.

[21] Kim S., Kim S. Y., Pribis J. P. (2013). Signaling of high mobility group box 1 (HMGB1) through toll-like receptor 4 in macrophages requires CD14.

[22] Fiuza C., Bustin M., Talwar S. (2002). Inflammation-promoting activity of HMGB1 on human microvascular endothelial cells.

[23] Chen Q., Guan X., Zuo X., Wang J., Yin W. (2016). The role of high mobility group box 1 (HMGB1) in the pathogenesis of kidney diseases.

[24] Brieland J. K., Jones M. L., Clarke S. J., Baker J. B., Warren J. S., Fantone J. C. (1992). Effect of acute inflammatory lung injury on the expression of monocyte chemoattractant protein-1 (MCP-1) in rat pulmonary alveolar macrophages.

[25] Standiford T. J., Kunkel S. L., Phan S. H., Rollins B. J., Strieter R. M. (1991). Alveolar macrophage-derived cytokines induce monocyte chemoattractant protein-1 expression from human pulmonary type II-like epithelial cells.

[26] Beck-Schimmer B., Schwendener R., Pasch T., Reyes L., Booy C., Schimmer R. C. (2005). Alveolar macrophages regulate neutrophil recruitment in endotoxin-induced lung injury.

[27] Tang D., Kang R., Zeh H. J., Lotze M. T. (2010). High-mobility group box 1 and cancer.

[28] Xia Q., Xu J., Chen H. (2016). Association between an elevated level of HMGB1 and non-small-cell lung cancer: a meta-analysis and literature review.

[29] Feng A., Tu Z., Yin B. (2016). The effect of HMGB1 on the clinicopathological and prognostic features of non-small cell lung cancer.

[30] Kang R., Tang D., Livesey K. M., Schapiro N. E., Lotze M. T., Zeh H. J. (2011). The receptor for advanced glycation end-products (RAGE) protects pancreatic tumor cells against oxidative injury.

[31] Zemans R. L., Colgan S. P., Downey G. P. (2009). Transepithelial migration of neutrophils: mechanisms and implications for acute lung injury.

[32] Kropski J. A., Fremont R. D., Calfee C. S., Ware L. B. (2009). Clara cell protein (CC16), a marker of lung epithelial injury, is decreased in plasma and pulmonary edema fluid from patients with acute lung injury.

[33] Serpa Neto A., Campos P. P., Hemmes S. N. (2017). Kinetics of plasma biomarkers of inflammation and lung injury in surgical patients with or without postoperative pulmonary complications.

[34] Taniguchi T., Kurita A., Kobayashi K., Yamamoto K., Inaba H. (2008). Dose- and time-related effects of dexmedetomidine on mortality and inflammatory responses to endotoxin-induced shock in rats.

[35] Chen F. F., Zhang D., Wang Y. L., Xiong B. (2013). Video-assisted thoracoscopic surgery lobectomy versus open lobectomy in patients with clinical stage I non-small cell lung cancer: A meta-analysis.

[36] Zarbock A., Eroglu A., Erturk E., Ince C., Westphal M. (2014). Ischemia-reperfusion injury and anesthesia.

[37] Baines M., Shenkin A. (2002). Use of antioxidants in surgery: a measure to reduce postoperative complications.

[38] King R. C., Binns O. A. R., Rodriguez F. (2000). Reperfusion injury significantly impacts clinical outcome after pulmonary transplantation.

